# Supplementary material for: Health Service Interventions for Intimate Partner Violence among Military Personnel and Veterans: A Framework and Scoping Review
Source: Int J Environ Res Public Health. 2022 Mar 17;19(6):3551. doi: 10.3390/ijerph19063551 (PMC8955703; doi:10.3390/ijerph19063551)
Supplement: Supplementary file 1 [file ijerph-19-03551-s001.zip › Supplementary Table S1.pdf]

Supplementary Table S1

*Example Search Strategy Conducted in PsycINFO Database*

| Step | Search Terms | No of Records |
|---|---|---|
| S1 | battered females/ or domestic violence/ or intimate partner violence/ | 21513 |
| S2 | (IPV OR "intimate partner violence" OR "partner violence" OR "spousal violence" OR "domestic violence" OR "intimate partner abuse" OR "partner abuse" OR "spouse abuse" OR "intimate partner victim*" OR "intimate partner perpetrat*" OR "family violence")ab,mh,ti. | 22811 |
| S3 | ((Abus* OR batter* OR violen* OR beat* or assault*) adj3 (perpetrat* OR victim* OR domestic OR partner* OR famil* OR spous* OR wom*n OR m*n OR wife OR wives OR husband* OR gender)).ab,ti. | 48376 |
| S4 | S1 or S2 or S3 | 51509 |
| S5 | military personnel/ or air force personnel/ or army personnel/ or coast guard personnel/ or marine personnel/ or military veterans/ or national guard personnel/ or navy personnel/ | 28344 |
| S6 | ("ex-military" OR veteran* OR military OR "active duty" OR "military personnel" OR army OR navy OR marines OR "air force" OR "armed forces" OR "military service personnel" OR "soldier").ab,mh,ti. | 52459 |
| S7 | S5 or S6 | 55355 |
| S8 | epidemiology/ or population/ | 53347 |
| S9 | prevalence.mp. | 125613 |
| S10 | (prevalence OR prevalent OR occurrence OR incidence OR epidemiol* OR population OR representative).ab,mh,ti. | 506520 |
| S11 | (random* NEAR/3 (select* or sampl*)).ab,ti. | 36502 |
| S12 | S8 or S9 or S10 or S11 | 538246 |
| S13 | Exp mental health services/ | 41545 |
| S14 | treatment/ or intervention/ or prevention/ | 159289 |
| S15 | ("Health*care setting*" or "Health*care service*" or "Health*care system*" or "Health*care centre*" or "Health setting*" or "Health service**" or "Health system" or "Health centre*" or “clinical setting” or “clinical service”).ab,mh,ti. | 89725 |

| | | |
|---|---|---|
| S16 | (Implement* or Treat* or program* or Interven* or Prevent* or Assess* or Screen* or "Veterans Health Administration" or VHA or Patient or provider or Clinic).ab,mh,ti. | 1978471 |
| S17 | S13 or S14 or S15 or S16 | 2016433 |
| S18 | S12 or S17 | 2234432 |
| S19 | S4 and S7 and S18 | 799 |
| S20 | limited S19 to (peer reviewed journal and english language) | 554 |

*Note.* ab = abstract, mh = mesh headings, ti = title.
