# Supplementary material for: Health Service Interventions for Intimate Partner Violence among Military Personnel and Veterans: A Framework and Scoping Review
Source: Int J Environ Res Public Health. 2022 Mar 17;19(6):3551. doi: 10.3390/ijerph19063551 (PMC8955703; doi:10.3390/ijerph19063551)
Supplement: Supplementary file 1 [file ijerph-19-03551-s001.zip › Supplementary Table S2 R1.pdf]

Supplementary Table S2. Characteristics of included studies (n = 19)

**Authors**	**Year**	**Country**	**Sample**	**Intervention Target**	**Intervention Type**	**Study Design**
Chaffin and Richter [21]	2002	U.S.	*n* = 254 dental practitioners	IPV Victimization	Training program (1 hour) for service providers; 'Prevention of Abuse and Neglect through Dental Awareness' (PANDA).	Pre/Post
Chermack et al. [18]	2019	U.S.	n = 180 veterans (92% men)	IPV Perpetration	Integrative / eclectic program; Motivational Interviewing , CBT, and telephone monitoring (Continuing Care).	RCT
Creech et al. [32]	2018	U.S.	*n* = 51 veterans (all men)	IPV Perpetration	Group CBT program; Strength at Home-Men (SAH-M)	Pre/Post
Creech et al. [29]	2020	U.S.	*n* = 20 veterans (all women), including *n* = 9 reporting IPV victimisation	IPV Victimization	Brief psychosocial program; Safe and Healthy Experiences (SHE) intervention	Open trial
Danitz et al. [30]	2019	U.S.	*n* = 23 service providers	IPV Victimization	Extended psychosocial program; Recovering from IPV through Strength and Empowerment (RISE)	Qualitative
Edwardsen et al. [22]	2011	U.S.	*n* = 73 service providers	IPV Victimization	Training program (7-hour) for service providers	Pre/Post
Iverson et al. [23]	2013	U.S.	*n* = 160 veterans (all women)	IPV Victimization	Case identification strategy; Hurt/Insult/Threaten/Scream (HITS) screening tool	Cross-sectional
Iverson et al. [25]	2015	U.S.	*n* = 80 veterans (all women)	IPV Victimization	Case identification strategy; Extended HITS (E-HITS) screening tool	Cross-sectional
Iverson et al. [28]	2018	U.S.	*n* = 774 veterans (all women)	IPV Victimization	Case identification strategy; 3-item Danger Assessment secondary risk screening tool	Retrospective chart review
Iverson et al. [26]	2020	U.S.	NA	IPV Victimization	Case identification strategy; Screening tools and supporting toolkit	Protocol for RCT

**Authors**	**Year**	**Country**	**Sample**	**Intervention Target**	**Intervention Type**	**Study Design**
Latta et al. [24]	2016	U.S.	*n* = 93 patients attending a VA women's health clinic	IPV Victimization	Case identification strategy; HITS screening tool	Cross-sectional
Love et al. [31]	2015	U.S.	*n* = 6 veterans (all men), and *n* = 20 mental health service providers	IPV Perpetration	Group CBT program; SAH-M	Pre/Post (veterans), and cross-sectional (service providers)
McIntyre et al. [27]	1999	U.S.	*n* = 127 patients attending a VA women's health centre	IPV Victimization	Case identification strategy; Trauma Questionnaire (TQ)	Cross-sectional
Schaffer [19]	2010	U.S.	*n* = 76 veterans (all men)	IPV Perpetration	Integrative / eclectic program; Group Batterer Intervention Program (BIP)	Pre/Post
Schaffer [20]	2016	U.S.	*n* = 131 veterans (all men)	IPV Perpetration	Integrative / eclectic program; Group BIP	Pre/Post
Taft et al. [16]	2013	U.S.	*n* = 6 veterans (all men)	IPV Perpetration	Group CBT program; SAH-M	Pre/Post
Taft et al. [14]	2014	U.S.	*n* = 9 veterans (all men) and intimate partners (all women)	IPV Perpetration / Victimization	Couples program; Strength at Home – Couples (SAH-C)	Pre/Post
Taft et al. [17]	2016 a	U.S.	*n* = 135 veterans/service members (all men)	IPV Perpetration	Group CBT program; SAH-M	RCT
Taft et al. [15]	2016 b	U.S.	*n* = 69 veterans (all men) and intimate partners (all women)	IPV Perpetration / Victimization	Couples program; SAH-C	RCT

Note. CBT = Cognitive-behavioral therapy; IPV = Intimate Partner Violence; RCT = Randomised control trial; U.S. = United States.
