# Supplementary material for: Health Service Interventions for Intimate Partner Violence among Military Personnel and Veterans: A Framework and Scoping Review
Source: Int J Environ Res Public Health. 2022 Mar 17;19(6):3551. doi: 10.3390/ijerph19063551 (PMC8955703; doi:10.3390/ijerph19063551)
Supplement: Supplementary file 1 [file ijerph-19-03551-s001.zip › Supplementary Table S3 R1.pdf]

Supplementary Table S3. Summary of study characteristics for randomised trials

Study	Intervention	Sample	Eligibility	Design	Key Findings	Major Caveats
Taft et al. (2016) [15]	SAH-C	n = 69 couples (male veterans or AD personnel and partners) from the U.S.	Couples eligible if one or both partners reported: (a) significant relationship distress according to self-report scales; or (b) high psychological IPV perpetrated by men according to standardised self-report scales.<br><br>Couples were excluded if: female partners reported recent use of violence that involved weapons or produced injuries or partner reports of fear; and/or male partners reported physical IPV in the past 6 months.	Couples randomly allocated to SAH-C or a SP condition involving minimal therapist intervention.<br><br>Outcome measures included Physical and Psychological IPV subscales of CTS-2, and the MMEA.<br><br>Measures administered at: pre-intervention; immediate post-intervention; 6-months post-intervention; and 12-months post-intervention.	Participants in SAH-C were around twice as likely to complete the intervention, when compared to SP.<br><br>Veterans and partners in SAH-C tended to report lower physical and psychological IPV perpetration when compared to SP, with effect sizes ranging from small to moderate. However, with only one exception these differences were not significant ($p < 0.05$).	(1) Most comparisons between SAH-C and the control condition were not significant and findings are equivocal.<br><br>(2) Small sample size for a prevention study (which typically require large numbers to detect modest effects), and the lack of significant effects may be attributed to low statistical power.

Taft et al. (2016) [17]	SAH-M	*n* = 135 male veterans or AD personnel from U.S, and *n* = 111 female partners (collateral informants).	Male veterans or AD personnel eligible if: (a) they provided partner contact consent; and (b) there were self-reports, collateral-reports, or court-reports of any male-to-female physical IPV over the previous 6-months, or severe physical IPV over the past 12-months, or an ongoing legal problem related to IPV.	Couples randomly allocated to SAH-M or an ETAU condition involving referrals to services inside and outside the VHA, continued assessment for IPV, and a check-in call between assessments.<br><br>Outcome measures included Physical and Psychological IPV subscales of the CTS-2, and MMEA. Measures administered at: pre-intervention; immediately post SAH-M (3 months after baseline for ETAU); and 3-months post SAM-M (6-months after baseline for ETAU).	SAH-M associated with significantly ($p < 0.05$) greater declines overall in scores on the Physical IPV and Psychological IPV subscales of the CTS-2, and Restrictive Engulfment subscale of the MMEA (but not other subscales), when compared to ETAU.<br><br>Follow-up analyses indicated significantly lower scores on the CTS-2 subscales in the SAH-M condition at post-treatment, although effects were reduced and were not significant at 3-month follow-up.	(1) More than 50% of participants in SAH-M were classified as non-completers.<br><br>(2) The sample included a small number of participants (*n* = 9) that did not self-report any physical IPV, but were included on the basis of court involvement or collateral reports of IPV.
Chermack et al. (2019) [18]	MI-CBT and MI-CBT+CC	n = 180 veterans (92% male) seeking substance use and/or mental health treatment from VHA.	Veterans seeking substance use and/or mental health treatment were eligible if they reported past-year: (a) substance use; and (b) physical aggression directed to partners or non-partners. The latter as operationalised by physical IPV and injury subscales from the CTS-2, and a modified version of the CTS-2 that was adapted to reference non-partner aggression.	Participants randomly allocated one of two active interventions (MI-CBT or MI-CBT+CC) or Enhanced Treatment as Usual (ETAU). The latter involved participation in standard VHA substance use and/or mental health treatment programs, along with a single session of brief advice to avoid aggression and review of referral information.<br><br>Outcome measures included a structured interview (using a calendar approach) based on CTS-2 Physical IPV and Injury subscales, and a Timeline Follow-Back	All conditions demonstrated significant decreases in Physical IPV over time, and there were low levels of violence disclosed across all groups and follow-up periods (10.9% of all participants reported Physical IPV towards partners and 2.9% reported Partner Injury over the entire 12-month post-intervention period).<br><br>In the context of limited variability, there was a significantly greater decreases in Physical IPV towards partners in the MI-CBT + CC condition,	(1) Measures of IPV were based entirely on participant disclosures and there were no collateral (e.g., partner) reports of violence.<br><br>(2) There low levels of IPV disclosed across all conditions and follow-up periods, and thus limited variability which could limit the ability to detect between-group effects.

measure for alcohol and drug use. Measures were administered at baseline and 3-months, 6-months, and 12-months post-intervention.	relative to ETAU. However, no other significant between-group differences were observed.	(3) Interventions were focussed on IPV and non-partner aggression, and not all participants reported violence directed towards partners.

Notes. AD = Active duty; CTS-2 = Conflict Tactic Scale – 2; ETAU = Enhanced Treatment as Usual; IPV = Intimate partner violence; MI-CBT = Motivational Interviewing – Cognitive Behavioural Treatment; MI-CBT+CC = Motivational Interviewing – Cognitive Behavioural Treatment plus Continuing Care; MMEA = Multidimensional Measure of Emotional Abuse; SAH-C = Strength at Home – Couples; SAH-M = Strength at Home – Men; SP = Supportive Prevention; U.S.= United States; VHA = Veteran Health Administration.
